# Pushing the Boundaries of Health Self-Management With Conversational AI

**DOI:** 10.3389/ijph.2026.1608975

**Published:** 2026-01-16

**Authors:** Enxhi Qama

**Affiliations:** 1 University of Lucerne, Lucerne, Switzerland; 2 Person-Centered Healthcare and Health Communication, Schweizer Paraplegiker-Forschung, Nottwil, Switzerland

**Keywords:** chronic conditions, conversational agents, patient education, prompt initiation, self-management

**The IJPH series “Young Researcher Editorial” is a training project of the Swiss School of Public Health.**

Self-management is the ability of individuals, supported by their networks and healthcare systems, to manage symptoms, treatments, and the social and emotional consequences of chronic conditions [1]. Self-management rarely comprises a fixed set of routines; instead it is shaped by relationships, constrained and enabled by context, and adjusted through trial and error. Core skills include interpreting bodily cues, adapting routines when circumstances change, and integrating health practices into the rest of life’s demands [2].

Against this background of adaptive, trial-and-error self-management, conversational AI tools such as ChatGPT are increasingly entering health-related contexts [3]. Building on their emerging use in other health contexts [4], individuals my turn to them not only for information, but also for open-ended exchanges during which they explore “what-if” scenarios through dialogue. Such exchanges can be regarded as a continuation of long-standing self-experimentation practices in self-management, although mediated by an external system whose suggestions must be interpreted cautiously. These dialogues include interaction on adjusting timing or types of medications, testing exercise routines, or adapting diet and daily activities, while monitoring outcomes to see what works best for their condition [2]. The difference is that the support would no longer come from a friend, family member, or clinician, but from an algorithm generating tailored responses in seconds. Early research suggests that such tools can support reflection, problem-solving, and even companionship for some users [5, 6].

To understand what this means for self-management, it is important to briefly clarify how conversational AI systems generate responses. For human-LLM interactions to be productive, people need to be able to ask the right questions and provide the right context. Conversational AI tools are powered by large language models (LLMs), which generate responses by predicting word sequences based on training datasets. They do not “know” things, but approximate answers shaped by their training data, rules, and user inputs [4]. This means that the relevance and reliability of outputs are influenced both by the underlying model and by how users frame their questions. In AI circles, this is linked to prompt engineering, the technical practice of crafting inputs (prompts) that guide an AI system toward more relevant and reliable results. A broader, and related concept is *prompt literacy*: the ability of the user to understand how an AI interprets prompts, to formulate effective questions, and to refine them based on responses [7]. These technical characteristics directly shape how AI can, and cannot, support self-management decisions in everyday life.

Within the context of chronic condition self-management, prompt literacy can be understood as having two complementary dimensions. One is a practical skill: users must frame questions about, for example, a chronic condition, requesting usable information, and refining follow-ups to obtain outputs that are relevant and easier to integrate into their routines. The other skill is interpretive: users must distinguish supportive AI outputs from professional medical advice. LLM recommendations will reflect the biases of their training data and should be critically appraised before users act upon them [8]. In this sense, prompt literacy extends digital health literacy into the space of human–AI interaction. While digital health literacy emphasizes searching and appraising online information, prompt literacy allows users to shape the interaction by co-producing information through dialogue.

Because self-management is shaped by social and structural conditions, these interactional skills also have implications beyond individual patients, more specifically, for public health. Digital health disparities stem not only from differences in access to devices or connectivity, but also from differences in skills [9]. Someone with strong prompt literacy skills might co-create a nuanced self-management plan with an AI tool, while someone without it may obtain only generic advice that is harder to put to use or even misleading. Risk of misinformation remains high, as outputs are shaped by training data of unknown quality that replicate biases. Targeted education and support for patients and also for family caregivers could help narrow this gap. Caregivers often take on substantial responsibilities and may act as intermediaries between patients and AI systems. Individuals with chronic conditions may benefit from community health programs that integrate prompt literacy, offer practical workshops, or embed training into rehabilitation curricula to prepare people before they leave structured care.

Prompt engineering could also be integrated into care. Most patient–AI interactions occur outside formal healthcare settings, where LLM use offers flexibility and privacy but also raises questions about accuracy, safety, and alignment with professional guidance [10]. Should care programs train patients in prompt literacy? Could AI-assisted self-management logs inform consultations or would that place new burdens on patients and providers? Research is needed on outcomes, clinician workload, patient–provider relationships, and the trustworthiness of AI-assisted recommendations.

Finally, there are limits to what AI in self-management can offer. While conversational agents can simulate empathy and offer a sense of presence [6], they cannot experience illness or assume responsibility for outcomes. Nor can they grasp context unless it is explicitly provided, something that requires user skill and vigilance. Viewed this way, prompt literacy extends digital health literacy into the space of human–AI interaction. Whereas digital health literacy emphasizes searching and appraising online information, prompt literacy focuses on shaping the interaction itself, by co-producing information through dialogue. When we examine self-management through this lens, we can see the need for interventions co-designed with patients to ensure AI tools are not only technically capable, but also socially and contextually aligned with the realities of living with chronic conditions. The future of self-management may depend less on automating decisions, and more on increasing individuals’ capacity to make accurate and adequate decisions.
